# Development, Construct Validation, and Normalization of a New Early Childhood Self-Regulation Assessment Scale

**DOI:** 10.1007/s10643-022-01310-9

**Published:** 2022-02-26

**Authors:** Wanda Boyer

**Affiliations:** Department of Educational Psychology and Leadership Studies, University of Victoria, 3800 Finnerty Road (Ring Road), P.O. Box 1700 STN CSC, Victoria, BC V8W-2Y2 Canada

**Keywords:** Early childhood self-regulation, Social constructivism, Construct validity, Assessment normalization, Exploratory sequential mixed methods, Grounded theory

## Abstract

Although there are many tools for assessing young children’s self-regulation according to varied conceptual definitions and purposes, the purpose of this study was to develop, validate, and norm a Self-Regulation Assessment Scale for Early Childhood (SASEC) for directly evaluating observed behaviors of young children in naturalistic play experiences within the normal preschool environment. An exploratory sequential mixed methods research design was used. The 315 participants included 153 parents and 15 educators for the qualitative component and 147 children ages 3–5 years for the quantitative component. The analytical steps of a *qualitative grounded theory research design were applied to* adult participant interviews and focus group discussions, which culminated in 12 scale items for measuring a child’s ability to initiate, modulate, and cease behaviors, tasks, or activities of varied complexities, social configurations, and limiting conditions. Children’s SASEC scores were assessed via video recordings of play behaviors in naturalistic settings. Based on factor analysis results, the SASEC items constitute a single construct. According to the results of hierarchical linear modeling and multiple linear regression, preschool children’s SASEC scores can be compared to the SASEC mean and standard deviation regardless of various demographic variables. Implications and recommendations for future work include having early childhood educators, child and youth care practitioners, counselors, parents and families, social workers, behavioral sciences researchers, and policy makers use the SASEC to measure young children’s self-regulation while developing or monitoring the efficacy of generalized enhancement programs and individualized treatment plans.

## Introduction

Children’s misbehavior and aggression are ongoing concerns (Poulou, 2015; Smith Watts et al., 2019) because of the long-term effects on society of inadequately addressing young children’s acquisition of self-regulation (Perry et al., 2018; Wesarg et al., 2020). Behavior problems emerging in early childhood often endure into adulthood and have been related to various aspects of parent–child and teacher–child relationships (Compagnoni et al., 2019; Graziano et al., 2016; Helm et al., 2020; Mason et al., 2020; Montroy et al., 2016; Neuhaus et al., 2020). In contrast, an early ability to self-regulate by controlling behavioral impulses has been correlated with a range of greater competencies in later life including cognitive and solution-focused planning abilities, moral and prosocial behaviors, academic achievement in mathematics, literacy, and vocabulary, fewer internalizing problems related to depression or anxiety, and healthier life choices related to food, exercise, and illicit drug use (Bégin et al., 2020; McCoy et al., 2019; Nofziger & Johnson, 2020; Perry et al., 2018; Robson et al., 2020; Sawyer et al., 2015).

The success factors of self-regulation acquisition in young children include supportive guidance by caregivers such as early childhood educators, parents, and community members (Bégin et al., 2020; Kosterman et al., 2019). However, early in this study, both early childhood educators and parents expressed concerns about their ability to provide appropriate behavioral guidance because, they noted, they “don’t really know what’s normal at this age” for their children’s development of self-regulatory skills. To address these concerns, early childhood educators, parents, and other adult caregivers need to be able to assess a child’s acquisition of self-regulation *relative to* what is typical for preschoolers.

There are challenges when assessing a child’s development of self-regulation due to varied conceptual definitions and purposes. Self-regulation assessments in the literature include (a) a tool developed for individualized early childhood health screening for the severity and dysregulation of attention, behavior, and emotional expression (Barbarin et al., 2020); (b) an assessment battery of tasks to discern kindergarten readiness (Bassett et al., 2012); (c) the Preschool Self-Regulation Assessment consisting of hot regulatory tasks that involve delay and emotional engagement (toy wrap, toy wait, snack delay, and tongue task), cool novel neutral tasks (the pencil tap, balance beam, and tower turn-taking), and three compliance tasks (tower clean-up, toy sort, toy return) to note strengths and weaknesses in preschooler’s self-regulation (Rademacher & Koglin, 2020); (d) the Parent–Child Challenge Task to study self-regulation displayed during dyadic patterns completing a puzzle with only parental verbal guidance (Lunkenheimer et al., 2017); (e) the Head-Toes-Knees-Shoulder task to measure behavioral regulation and problematic disruptive behavior and self-regulation associated with error-related negativity, feedback-related negativity, and error positivity or conscious processing or evaluation of errors after they have occurred (Coelho et al., 2019; Grabell et al., 2017; Graziano et al., 2016); (f) the Go/No-go tasks that measure attention, impulsivity, and inhibition (Berti & Cigala, 2020); (g) the Duck Task, which measures attentional skills by having a child select and put a card depicting a yellow-beaked duck beside a pen and also selecting from cards also including a duck with a white beak (Grob et al., 2013); (h) the Gift Delay task, which measures inhibitory responses that has a child delay responses while watching a gift being gift wrapped, delay asking about the gift, and delay touching the gift before they are told that they can unwrap the gift (Grob et al., 2013); (i) the Child Self-Regulation and Behavior Questionnaire, which is a 33-item educator or parent assessment of their opinions about a child’s cognitive, behavioral, and emotional self-regulation and other facets of social development (Howard & Melhuish, 2017); and (j) the Preschool Situational Self-Regulation Toolkit, which engages a child in researcher-selected activities that are intended to reflect potential experiences a child may have in preschool, such as a memory card game, a curiosity box guessing game, and an obstacle course (Howard et al., 2019). These self-regulation tools are constructed by researchers and are either based on evaluating a child’s performance in artificial scenarios or based on generalized responses and opinions from an educator or parent, rather than directly evaluating a young child’s observed behaviors in naturalistic play experiences within the normal preschool environment.

The present research study contributes to the field by addressing the above concerns and challenges for early childhood educators and parents by sequentially answering three research questions. The first research question was: What are the perspectives of early childhood educators and parents about what early childhood self-regulation is and how to assess the development of self-regulation of preschool children involved in naturalistic play experiences within the normal preschool environment? To begin answering this first research question, a qualitative grounded theory research design (Creswell & Guetterman, 2019) was used to thematically categorize the perspectives of the early childhood educators and parents into a core phenomenon and key propositions about early childhood self-regulation.

Based on the qualitative research results on the perspectives of the early childhood educators and parents about early childhood self-regulation, the researcher developed a 12-item Self-Regulation Assessment Scale for Early Childhood (SASEC). Contingent on the development of the SASEC, the second research question was: Are the items of the SASEC measure sufficiently inter-correlated such that the SASEC measures early childhood self-regulation as one construct? To answer this second research question, a quantitative correlational research design was used. Specifically, an exploratory factor analysis was performed.

Once the SASEC was developed and shown to measure early childhood self-regulation as a unitary construct, the third research question addressed in this study was: How can the SASEC be used to determine a self-regulation percentile score for a child in the early childhood age range? To answer this third research question, normalization descriptive statistics were calculated and then supported by a correlational research design. Specifically, the mean and standard deviation of SASEC total scores were first determined for the 3- to 5-year-old age range of children. Then, correlational analyses were performed to test for significant relationships between the SASEC total score as a dependent variable and the preschool the children attended as well as the children’s age, gender, years of parental experience, birth order, number of siblings, self-preferred ethnic designation of the child’s family, and estimated family annual income.

A hierarchical linear model was used to test the relationship between SASEC total score and a child’s preschool. Subsequently, a multiple linear regression analysis was used to test the relationship between SASEC total score and the remaining demographic variables.

Taken together, the qualitative and quantitative research designs used to answer the three research questions comprise the exploratory sequential mixed methods research design (Creswell & Guetterman, 2019) used in this study. Based on the three research questions, the purpose of this exploratory sequential mixed methods research study was to:

1. Develop a self-regulation assessment scale for preschoolers that (a) reflects early childhood educator and parental experiences of and beliefs about the preschool environment, (b) can be quickly administered within a busy preschool day, and (c) is based on observed behaviors in naturalistic play experiences within the normal preschool environment;
2. Measure the construct validity of the assessment scale, specifically that the items of the scale measure early childhood self-regulation as one construct; and
3. Determine norms on the assessment scale for the preschool age range so that early childhood educators, parents, and other adult caregivers can compare a child’s acquisition of self-regulation to what is typical for preschoolers.

This paper first explores the participants and research designs that led to the development, validation, and normalization of the SASEC. The paper then discusses the implications of the normalization results for early childhood educators, parents, counsellors, other caregivers, policymakers, and researchers as they use this assessment scale to support the development of children’s acquisition of self-regulation.

## Method

### Research Design Overview

In this study, an exploratory sequential mixed methods research design was necessary to achieve all three elements of the purpose of the study, which was to develop, validate, and normalize a new early childhood self-regulation assessment scale. In the qualitative phase of this research design, a grounded theory research method (Creswell & Guetterman, 2019) was necessary to obtain and thematically categorize the perspectives of early childhood educators and parents of preschoolers into a core phenomenon and key propositions about early childhood self-regulation. Based on the core phenomenon and key propositions from the early childhood educators and parents of preschoolers, the researcher was able to develop a 12-item assessment tool, the SASEC, for assessing early childhood self-regulation based on observed behaviors in naturalistic play experiences within the normal preschool environment. In the quantitative phase of the research design, the SASEC was administered to a population of preschoolers, and correlational research methods were used on the results. Specifically, construct validity was measured with an exploratory factor analysis to validate that the SASEC measured early childhood self-regulation as a single construct (American Educational Research Association, American Psychological Association, & National Council on Measurement in Education, 2014), and then hierarchical linear modeling, multiple linear regression, and descriptive statistics were used to determine normalization results for the 3- to 5-year age range. Whereas the qualitative phase was necessary to develop the SASEC, the quantitative phase was necessary to enable the comparison of any given child’s SASEC total score with the expected SASEC total score range for preschoolers.

### Participants

There was a total of 315 participants in this study, including 147 children ages 3–5 years from 143 families, 153 of the children’s parents, and 15 early childhood educators across seven preschool sites. The sites were selected via purposeful sampling according to the following criteria: the site must serve children ages 3–5 years, provide preschool education experiences, have a mission statement and guiding principles, and be located in the North American Pacific Rim. Within the sites, the 147 out of 164 children (90%) and their parents and early childhood educators gave ongoing informed consent to participate in the study, including partaking in interviews, focus groups, and video-taped naturalistic play experiences. Of the 164 children at the seven preschool sites, only 17 were excluded from this study, mostly due to parents indicating that they did not have the time to participate in the qualitative component of the research. When video-taping the 147 child participants, the excluded children were given the option of being in the background or moving to a different location, such as attending a different center, task, or activity. The author’s institutional research ethics board approved the ethics application for this study.

The preschool programs were co-educational and varied in structure, with full-day and half-day programs and both mixed and homogeneous age groups. The preschools included parent co-operatives and settings without parent involvement, urban and rural settings, and settings that were religious, spiritual, and neither. Within the preschool programs, the 15 early childhood educators ranged in age from 28 to 56 years with a mean age of 45. Their self-preferred ethnic designations included Chinese (Mandarin speakers), European ancestry, First Nations, and multi-ethnic. The early childhood educators’ years of service ranged from 1.5 to 23 years with a mean of 12 years, and their training ranged from 1 to 5 years with a mean of 2.5 years of training.

The families of the children in the study consisted of 116 families (81%) with self-preferred ethnic designations of European ancestry and 27 families (19%) with other self-preferred ethnic designations of Chinese, Filipino, First Nations, Japanese, Métis, Mexican, and South East Asian, as well as self-preferred multi-ethnic designations that included African-American, Arabic, Brazilian, Chinese, Eurasian, Filipino, Ghanaian, Hispanic, Israeli, Japanese, Maltese, South Asian, South East Asian, and South African. Of the 143 families, 8 were single parent families (7 single mother families and one single father family). Based on employment data provided by the parents, there were 102 middle income families (71%) and 41 low income families (29%).

The parents of the children in this study had mean ages of 34 (SD = 5.9) for mothers and 35 years (SD = 5.6) for fathers. Of the children’s parents, 153 parents (140 mothers and 13 fathers) participated in interviews and focus groups. The parents had an average of 6.03 years of parenting experience (SD = 3.06).

The 147 children in the study had a mean age of 4.1 years (SD = 0.65) and were comprised of 90 girls and 57 boys. There were 83 first born children (56%). There were 33 children with 0 siblings, 86 with 1 sibling, 23 with 2 siblings, 4 with 3 siblings, and 1 child with four siblings.

### Data Collection for the Qualitative Phase

The first four data sources for the qualitative phase were text transcripts of 45- to 60-minute audio-taped interviews and 90- to 180-minute audio-taped focus group sessions with the parents and, separately, with the early childhood educators. The intention of performing individual interviews and focus group sessions was to gather, for answering the first research question, the perspectives of the early childhood educators and the parents of preschoolers about the definition and assessment of early childhood self-regulation within the normal preschool environment. The interviews and focus group sessions included discussion of 18 open-ended individual interview questions and 12 open-ended focus group questions, which were supported by the literature (Bronson, 2000; Kemple et al., 1997; Kochanska et al., 2001; Kopp, 1982). Specifically, within the theoretical framework of social constructivism (Vygotsky, 1966/1979), Kopp (1982) defined self-regulation in young children as the ability to initiate, modulate, or cease behaviors to comply with caregiver standards. Via social interactions, young children are able acquire the inhibitory and effortful control, attentional focus and flexibility, and working memory (Lipsey et al., 2017; McClelland & Cameron, 2012) needed to initiate or begin activities at the request of caregivers, to modulate or modify their responses to an activity when requested, and to cease or stop behaviors when asked by caregivers. The 18 questions on self-regulation skills acquisition of 3- to 5-year-olds thematically covered child temperament, the processes to initiate, modulate, and cease activities, and strategies adults use to support the growth of self-regulation. The 12 focus group questions involved the focus groups in synergistically discussing the definition of self-regulation, the effects of nature and nurture on the acquisition of self-regulatory skills, attitudes contributing to self-regulation, developmental differences in self-regulatory skills across age groups, and the importance of self-regulation to the caregiving process of early childhood educators and parents.

A fifth data source was the official preschool documents for each preschool that described the preschool’s policies and code of conduct. These documents were included because it was expected that their content would include guidelines for initiating, modulating, and ceasing behaviors according to early childhood educator and parental expectations such that these documents would contain written information on the early childhood educators’ and parents’ perspectives about early childhood self-regulation.

### Data Collection for the Quantitative Phase

Once the 12-item SASEC was developed using qualitative analysis on the data for the qualitative phase, the children in the study were rated with the SASEC. The data for these ratings consisted of video-taped 30-minute naturalistic play experiences for each of the child participants. These play experiences included the child’s interactions with early childhood educators, parents, and other children, and they included a variety of learning and living contexts such as preschool center stations and activities, indoor and outdoor free play and organized games, meal and snack times, and field trips. The intention of video-taping naturalistic play experiences of the child participants was to enable the rating of the child participants with the SASEC. The item-level SASEC scores were the input data for measuring construct validity of the SASEC with an exploratory factor analysis to answer the second research question. The SASEC total scores were the input data for calculating the descriptive statistics as well as for the dependent variable of the hierarchical linear modeling and multiple linear regression correlational analyses to answer the third research question.

Demographic data about the children was also collected using forms completed by the parents. Demographic data included the child’s age, gender, number of siblings, and birth order. Further demographic data about the child’s parents included the ages of the mother and father, marital status, number of years of parental experience (i.e., age of their oldest child), self-preferred ethnic designation of the child’s family, and employment descriptions for the mother and father. The parents’ employment descriptions were mapped to employment categories given by Statistics Canada (2020a) to transform them into 2018 median weekly national income values for mothers and fathers in the age range 25–54 years. These were summed per family and multiplied by 52 weeks per year to produce the estimated family annual income, which was then compared to the 2018 median annual family income defined by Statistics Canada (2020b). A middle-income family was defined as “earning between 75 and 200% of the median national income” (Organization for Economic Co-operation and Development [OECD], 2019, p. 13) for 2018, and low-income families were those with income less than 75% of the median annual family income. These demographic data elements were used as input for the independent variables in the hierarchical linear modeling and multiple linear regression correlational analyses to answer the third research question.

### Data Analysis for the Qualitative Phase

The qualitative data was analyzed using the grounded theory research method. This method consisted of the following three steps: open coding, axial coding, and selective coding (Creswell & Guetterman, 2019).

In the open coding step, the grounded theory researcher began with a form of content analysis articulated by Sandelowski (2000) to stay close to the data, to understand what happened and to remain true to the knowledge conveyed by the participants, while summarizing the data. The grounded theory researcher segmented the summarized data into categories, dimensionalized properties, and examples. The analysis consisted of employing a ‘zig-zag’ method of comparative and inductive procedures to achieve saturation of categories with the intent to ‘ground’ the categories in the data, eliminate redundancy, and develop evidence for the categories (Creswell & Guetterman, 2019).

In the axial coding step, the grounded theory researcher selected one open coding category and positioned it in the center of the process, labeling it the core phenomenon. From the core phenomenon, the axial coding proceeded with the grounded theory researcher classifying the remaining open coding categories according to (a) causal conditions, which are the categories of conditions that influence the core category; (b) context, which provides the specific conditions that influences the strategies; (c) intervening conditions, which are the general contextual conditions that influence strategies; (d) strategies, which are the specific actions or interactions resulting from the core phenomenon, and (e) consequences, which are the outcomes of employing the strategies (Creswell & Guetterman, 2019). As the result of axial coding, the grounded theory researcher developed a logical paradigm, a visual picture, of the theory of self-regulation for early childhood.

In the selective coding step, the grounded theory researcher wrote a story line that interconnected the categories in the axial coding paradigm (Creswell & Guetterman, 2019). The story examined the factors that influenced the phenomenon leading to specific strategies with certain outcomes. Ultimately, the grounded theory researcher generated propositions and ideas to guide the formulation of a self-regulation assessment tool for early childhood.

### Data Analysis for the Quantitative Phase

Based on the grounded theory research, the researcher formulated the 12-item Self-Regulation Assessment Scale for Early Childhood (SASEC). The construct validity of the SASEC was measured with exploratory factor analysis, and Cronbach’s alpha was used to measure reliability. The researcher then calculated normalization information in the form of means and standard deviations for each SASEC item score and the SASEC total score. A no-predictors hierarchical linear model was used to test whether or not there was a significant difference in SASEC total scores across preschool sites (Level 2). Based on there being no significant difference across preschool sites, multiple linear regression was then used to measure whether there were any significant relationships between the SASEC total score and any of the following demographic variables (Level 1): age, gender, years of parental experience, birth order, number of siblings, self-preferred ethnic designation of the child’s family, and estimated family annual income.

## Reliability

The 147 children’s 30-minute video-taped observations were evaluated with the 12-item SASEC by the both the researcher and a research assistant. Inter-rater agreement was 89.63% based on equality comparison of all 12 item scores for all 147 SASEC assessments. From the 1764 item scores, the researcher and the research assistant then discussed all 183 item score disagreements to produce revised consensus scores prior to quantitative analysis of the validity and reliability of the SASEC.

## Results

Based on the exploratory sequential mixed methods research design, the qualitative results are presented in a first section, followed by the quantitative results. The qualitative results of the grounded theory research method consist of three steps of coding results that culminated in the information necessary to develop the SASEC. The quantitative results consist of construct validity and reliability measurements of the SASEC; normalization information, including the mean and standard deviation of SASEC total score for 3- to 5-year-old children; a hierarchical linear model comparing SASEC total scores across preschool sites; and a multiple linear regression analysis comparing the SASEC total score with several demographic variables.

### Qualitative Development of the SASEC

In the first step of the grounded theory research, open coding, the results from the voices of the participant parents and early childhood educators were first synthesized into five descriptive categories: (a) definitions of self-regulation, (b) skills contributing to self-regulation, (c) explanations of how self-regulation is acquired, (d) explorations of the caregiver role, and (e) parent and early childhood educator identification of the need for support. The participants *defined* self-regulation for preschool children in various manners that were consistent with the definition from one parent, who said it was defined as “learning how to control themselves in different contexts and with different people.” The participants then highlighted eight dimensionalized *skills* that contribute to self-regulation, which are presented in Table 1 along with example quotes. As shown in the third column of Table 1, the self-regulation skills span the spectrum of developmental areas, including physical, cognitive problem-solving, linguistic, social, emotional, and moral classifications. Next, the participants explained that *self-regulation is acquired* through “adult guidance,” “experiences with natural consequences for misbehavior,” being given “opportunities to practice empathy,” and “direct teaching of skills to resolve conflicts.” The participants then described the *caregiver role* as being a “role model”, “self-knowing” and a purveyor of a “set of tools or strategies to monitor [one’s] behavior.” To take on their caregiver role, participants *identified the need for support* in the form of understanding the developmental progression of self-regulation skills as well as how to synchronize developmentally appropriate strategies of early childhood educators and parents to meet individual children’s needs. They also highlighted that their own self-regulatory responses to “stress, time limitations, and my child’s behavior have a huge impact” on the self-regulation of their children. They noted that they supported self-regulation through expression of our “own individual values and beliefs” and therefore have “a need for ongoing education about self-regulation.” As one parent elaborated, “I need to know about [the child’s] development and [how to structure environments] where my child can grow through play.” Other early childhood educators and parents advocated “direct teaching of skills” that enable children to articulate their personal emotions (“use your words”) and to resolve conflicts by discussing with children the “different expectations for appropriate behaviors in different contexts.”

**Table 1:** Skills contributing to self-regulation in early childhood

Dimensionalized skill	Example quote	Developmental area
Constructively using physical energy	“to learn that exercise and fresh air is good for them and can help them channel their energy and eliminate the need to be impulsive or anxious” (Parent Interview)	Physical
Effortful control	“spending less time being mad or frustrated or not doing what you are supposed to do so that you can find the joy in life” (Parent Focus Group)	Cognitive problem-solving
Stability/Consistency	“knowing and establishing certain schedules and activities you can count on like when to eat and sleep or take a break” (Parent Interview)	Cognitive problem-solving
Communication	Expressing “needs and wants in clear non-violent language” (Early Childhood Educator Focus Group)	Linguistic
Patience	“practicing the ability to wait your turn.” (Early Childhood Educator Interview)	Social
Optimism	“A sense of hopefulness” (Parent Focus Group)	Emotional
Controlling reactions to events	“feeling more confident, like ‘Hey, I did it, I didn’t cry or shout. I’m very brave.’” (Parent Interview)	Emotional
Empathy	as a means of “helping children stop and think of others before taking things personally” (Early Childhood Educator Interview)	Moral

#### Axial Coding Results

**Fig. 1:**
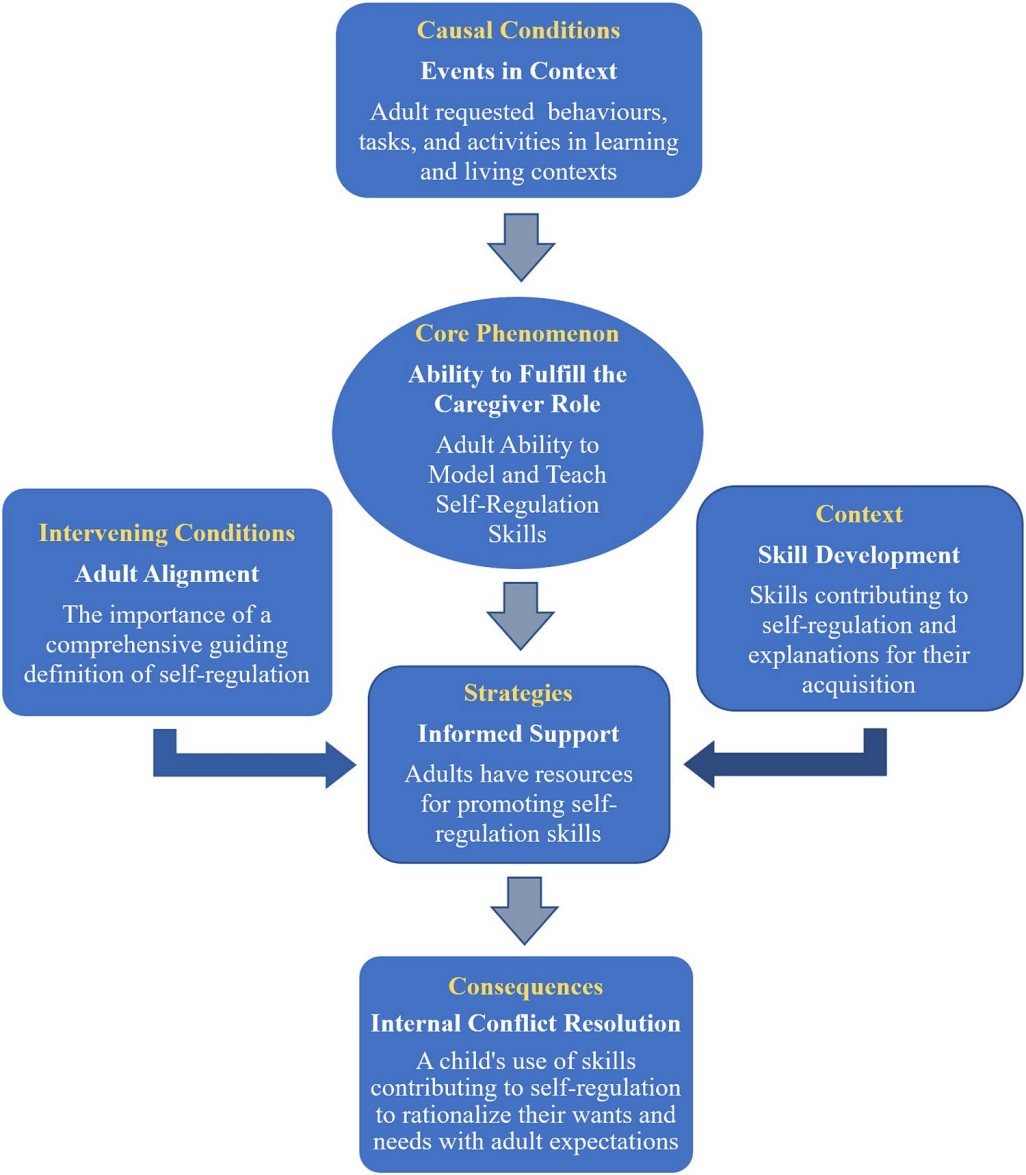
Axial coding of parent and educator perspectives on children's self-regulation

The results of the axial coding are presented in Figure 1. In the center of the process, the core phenomenon is the ability of adults to fulfill the caregiver role. The adult participants in this study were most concerned with their ability to model and teach self-regulation skills to the child or children in their care. The causal conditions leading to their concerns were the events or opportunities for a child in their care to demonstrate self-regulation skills in various learning and living contexts. The adult participants indicated that they needed support to fulfill the caregiver role of promoting self-regulation skills. A general intervening condition that adult participants found important was a clear and comprehensive definition of self-regulation. Adult caregiver alignment with a high precision definition of self-regulation can guide and influence all strategies for promoting self-regulation skills. As more specific contextual conditions that can affect the strategies of informed support, the adult participants highlighted specific skills contributing to self-regulation and a need for explanations of how those skills can be acquired. Based on their having the informed support necessary to help fulfill their caregiver role, adult participants expected as the outcome that their child would learn the self-regulation skills needed to help resolve the internal conflict between their wants and needs and adult expectations.

#### Selective Coding

The results of the selective coding consist of a narrative and a set of key propositions related to the axial coding paradigm. According to the adult participants in this study, a key aspect of fulfilling the caregiver role is the ability to model and teach self-regulation skills. However, they noted that they need more support to be successful. They identified a need for knowledge of specific skills contributing to self-regulation as well as explanations for how those specific skills are acquired. More generally, they noted a lack of a comprehensive guiding definition for self-regulation. Looking across the content from focus groups and interviews, individual adults anecdotally recognized parts of but not a whole, comprehensive definition of self-regulation that accounts for: (a) initiating, modulating, and ceasing as distinct notions; (b) differences in the degree of difficulty or the social configuration; and (c) the effects of limiting conditions.

This observation about the adults’ need of a comprehensive, guiding definition of self-regulation led to the following selective coding propositions related to modeling and teaching scenarios for early childhood development of any specific self-regulation skill:

1. Initiating, modulating, and ceasing are distinct and fundamental aspects that must each be emphasized.
2. Skills can be developed incrementally through variations of complexity, such as the degree of difficulty or the social configuration of a behavior, task, or activity.
3. Skills can be refined based on limiting conditions such as at specified time, speed, or location, or with specified materials.

These propositions guided the formulation of the 12-item Self-regulation Assessment Scale for Early Childhood (SASEC). Specifically, four items were devoted to each of initiating, modulating, and ceasing a behavior, task, or activity, and each subset of four items included variations of complexity, social configuration, or limiting conditions. The items of the SASEC appear in Appendix A. The measurement of each SASEC item was specified to be a 5-point Likert scale based on the extent to which a child is observed to self-regulate in the situation specified by the item. Verbal frequency expressions for each rating from 1 to 5 were aligned with semantics documented by Bocklisch et al. (2012). Further details about the verbal frequency expressions and operationalized definitions of the ratings appear in Appendix B. The SASEC total score was defined to be the sum of the numeric ratings on the 12 items.

### Validation and Reliability of the SASEC

The construct validity of the SASEC was measured with exploratory factor analysis. The efficacy of the factor analysis was established based on a high Kaiser–Meyer–Olkin (KMO) measure of sampling adequacy (0.943) and a statistically significant result on Bartlett’s test of sphericity (*p* < 0.001). The eigenvalues from the exploratory factor analysis revealed that the SASEC measured a single factor, as there was a high eigenvalue (10.761) for one factor and eigenvalues below one (0.328 and lower) for higher numbers of factors. The factor loadings of each SASEC item were high, as shown in Table 2.

**Table 2:** Results from a factor analysis of SASEC items

SASEC item	Factor loading
1	**0.948**
2	**0.949**
3	**0.948**
4	**0.958**
5	**0.959**
6	**0.956**
7	**0.939**
8	**0.941**
9	**0.924**
10	**0.950**
11	**0.945**
12	**0.946**

*N* = 147. The extraction method was principal component analysis with no rotation since the SASEC was found to measure a single unified factor. Factor loadings above .30 are in bold

The reliability (internal consistency) of the SASEC was found to be high, with a Cronbach’s alpha of 0.990. This result indicated that the items in the SASEC were closely correlated, which can be seen in the inter-item correlation matrix appearing in Table 3.

**Table 3:** SASEC inter-item correlation matrix

	1	2	3	4	5	6	7	8	9	10	11	12
1	–											
2	**0.957**	–										
3	**0.920**	**0.938**	–									
4	**0.919**	**0.943**	**0.933**	–								
5	**0.905**	**0.886**	**0.887**	**0.877**	–							
6	**0.888**	**0.889**	**0.879**	**0.918**	**0.950**	–						
7	**0.855**	**0.867**	**0.886**	**0.903**	**0.907**	**0.911**	–					
8	**0.887**	**0.868**	**0.892**	**0.880**	**0.923**	**0.894**	**0.883**	–				
9	**0.835**	**0.848**	**0.838**	**0.845**	**0.869**	**0.871**	**0.879**	**0.851**	–			
10	**0.876**	**0.862**	**0.868**	**0.886**	**0.898**	**0.900**	**0.851**	**0.875**	**0.892**	–		
11	**0.845**	**0.847**	**0.871**	**0.888**	**0.889**	**0.885**	**0.871**	**0.872**	**0.901**	**0.935**	–	
12	**0.882**	**0.872**	**0.862**	**0.885**	**0.889**	**0.876**	**0.854**	**0.871**	**0.877**	**0.951**	**0.936**	–

*N* = 147. Correlation values in bold indicate *p* < .01

### Normalization of the SASEC

For the 147 child participants, the mean SASEC total score was 24.17, and the standard deviation was 12.493. Table 4 shows these results along with means and standard deviations for each of the 12 items of the SASEC. The results of the no-predictors hierarchical linear model indicated that no further development of a multilevel model was warranted because the SASEC total score intercepts did not vary significantly across preschool sites (σ^2^ = 10.1738, Wald Z = 1.073, *p* = 0.283), even when the *p*-value is halved as per the recommendation of Heck et al. (2014). The intraclass correlation coefficient (ICC) indicated that only 6.485% of SASEC total score variance lies between preschool sites. While an ICC value up to 0.065 indicates that differences between the groups is not meaningful (Vajargah & Nikbakht, 2015), there was significant variance among individual children at each of the preschool sites (σ^2^ = 146.7088, Wald Z = 8.391, *p* < 0.001). According to the results of a multiple regression analysis appearing in Table 5, these variations among children were not due to any of the demographic variables.

**Table 4:** SASEC total score and per-item means and standard deviations

SASEC item	*M*	*SD*
1	2.08	1.120
2	2.01	1.092
3	2.00	1.104
4	2.01	1.104
5	2.05	1.119
6	2.02	1.101
7	2.04	1.085
8	2.06	1.106
9	1.96	1.066
10	1.95	1.100
11	1.96	1.097
12	2.02	1.101
Total	24.17	12.493

*N* = 147

**Table 5:** Regression coefficients of demographic variables on SASEC total score

Variable	*B*	*SE*	*t*	*p*	95% CI
Constant	20.079	8.101	2.479	0.014^*^	[4.062, 36.097]
Age	0.159	1.899	0.083	0.934	[− 3.596, 3.913]
Gender	0.988	2.232	0.443	0.659	[− 3.425, 5.402]
Ethnic	− 4.499	2.669	− 1.685	0.094	[− 9.776, 0.779]
Number of siblings	− 0.857	1.851	− 0.463	0.644	[− 4.517, 2.802]
Birth order	5.725	3.130	1.829	0.070	[− 0.464, 11.914]
Parent experience	− 0.819	0.717	− 1.142	0.255	[− 2.236, 0.598]
Family income	0.000	0.000	0.187	0.852	[0.000, 0.000]

*N* = 147. *p < .05

## Discussion

In this study, we used an exploratory sequential mixed methods research design to explore the problem of how early childhood educators, parents, and other adult caregivers could easily assess a young child’s acquisition of self-regulation skills in comparison to what is typical for preschoolers. Due to the exploratory nature of this research study, there were no expectations of the results that could be deemed as conflicting with the actual results. In the qualitative phase of the research design, the first element of the study’s purpose was attained via the development of a 12-item Self-Regulation Assessment Scale for Early Childhood (SASEC) based on applying the analytical steps of a grounded theory research design to the early childhood educators’ and parents’ interviews and focus group discussions about the definition and assessment of early childhood self-regulation according to the social constructivist epistemology. The insights and examples from their discussions about the dynamism of the preschool environment culminated in the notion of assessing varied special conditions in which a young child requires or further refines their self-regulatory capabilities. In the quantitative phase of the research design, the second and third elements of the study’s purpose were attained via the validation of and the determination of norms on the SASEC for the preschool age range.

### Study Implications

An important *theoretical implication* for the field of early childhood education is that the factor analysis of the SASEC from the quantitative phase of the research design not only provides direct empirical support for the social constructivist definition of self-regulation from Kopp (1982) but also supports expanding the social constructivist definition of self-regulation based on the parameters identified in the qualitative grounded theory results and represented in the 12 SASEC items. Specifically, *self-regulation in young children is.*

the ability to comply with caregiver standards in naturalistic settings by initiating, modulating, and ceasing behaviors, tasks, or activities with varied difficulty levels, social configurations, and limitations on time, speed, location, or materials.

From the quantitative phase results related to normalization, a first *practical implication* for early childhood educators and parents is that it is typical in early childhood for self-regulation behaviors to manifest infrequently or occasionally (a rating of 2). Adult caregivers may take some measure of comfort from knowing that there is nothing inherently wrong with a preschool child that doesn’t often or always self-regulate. Rather, the adult caregiver’s direct engagement and attunement with the preschool child is essential to the child’s acquisition of self-regulation skills (Erdmann & Hertel, 2019). Moreover, a second *practical implication* for early childhood educators, parents, and policymakers is that, according to the results of the correlational analyses in this study, a child’s SASEC total score can be directly compared with the SASEC total score mean and standard deviation *without adjustment* for age, gender, years of parental experience, birth order, number of siblings, self-preferred ethnic designation of the child’s family, and estimated family annual income, provided that the child and the child’s family are within the parameter ranges of the participants in this study.

A final implication of this research study is of both theoretical and practical importance. The SASEC has been developed with the perspectives of early childhood educators and parents for use in evaluating observed behaviors of young children in naturalistic play experiences within the normal preschool environment. The validation and normalization of the SASEC enable it to be used to measure the efficacy of both generalized self-regulation enhancement programs developed by behavioral sciences researchers, such as programs surveyed by Shiu et al. (2020), as well as individualized self-regulation treatment plans by early childhood educators, child and youth care practitioners, counselors, parents and families, and social workers. In turn, policymakers will be able to use the SASEC to form an evidentiary basis for setting standards and selecting enhancement programs for early childhood self-regulation.

### Limitations and Future Work

The child participant sample included 61% girls, which was more than the national proportion of 51% (Statistics Canada, 2017). The median of the estimated annual family income of families in this study was only 91% of the national median annual family income (Statistics Canada, 2020b). In the area of self-preferred ethnic designation, the participant sample had limited diversity. The participant sample also was limited to parents and children involved in preschool programs. Furthermore, while the quantitative analysis focused on children in the range of 3 to 5 years of age, the qualitative analysis that led to the formulation of the SASEC included the views of parents who had not only preschool children but also early childhood school-aged children. Therefore, it is reasonable to assume that the construct measured by the SASEC may also be applicable to children in the kindergarten to grade three age range. Based on these limitations of the participant sample, future work should expand the base of data available for updating normalization results of the SASEC.

Future work could also seek to measure convergent or discriminant validity between the SASEC and other self-regulation assessment tools. However, the comparison may be challenging because the other assessment tools, including those discussed in the introduction, have been identified as reflecting a narrow conceptual focus (McCoy, 2019), rather than encompassing the full social constructivist definition of self-regulation. These other self-regulation assessment tools also reflect a larger range of skills than operationalized, emphasize only challenges in self-regulation rather than measuring self-regulatory abilities, or are predicated on a child’s receptive language skills (McCoy, 2019). Furthermore, rather than measuring self-regulation in naturalistic settings, these other self-regulation tools are used in settings and with people who are not familiar to the children, thereby failing to achieve ecological validity (McCoy, 2019). As such, these other self-regulation assessment tools are not linked to self-regulation outcomes as a way to “develop more informed interventions” (McCoy, 2019, p. 69).

Future work with the SASEC can also focus on developing and measuring the efficacy of interventions that target enhancement of self-regulation skills, including the ability to initiate, modulate, and cease behaviors, tasks, and activities in situations having varied levels of complexity, social configurations, and limiting conditions. With generalized self-regulation enhancement programs and individualized self-regulation enhancement treatment plans that are *empirically proven* to improve self-regulation skills, early childhood educators, child and youth care practitioners, counselors, parents and families, social workers, behavioral sciences researchers, and other policymakers will be using evidence-based practices to help ensure behavior problems emerging in early childhood do not endure.

## Data Availability

N.A.

## Appendix A

### Self-Regulation Assessment Scale for Early Childhood (SASEC)

Child’s Name and Assessment Date:                                 Total Score:

**Table:**

**1**	**2**	**3**	**4**	**5**	1. How does the child respond when asked to do a familiar, routine behavior, task, or activity?
**1**	**2**	**3**	**4**	**5**	2. How does the child respond when asked to do a challenging behavior, task, or activity?
**1**	**2**	**3**	**4**	**5**	3. How does the child respond when asked to do a behavior, task, or activity with a partner or small group?
**1**	**2**	**3**	**4**	**5**	4. How does the child respond when asked to do a behavior, task, or activity with a large group or the whole class?
**1**	**2**	**3**	**4**	**5**	5. How does a child respond when asked to do or continue doing a behavior, task, or activity in a set location?
**1**	**2**	**3**	**4**	**5**	6. How does a child respond when asked to do or continue doing a behavior, task, or activity with limited materials?
**1**	**2**	**3**	**4**	**5**	7. How does a child respond when asked to do or continue doing a behavior, task, or activity slowly or quickly, or with lesser or greater speed, or within a time limit?
**1**	**2**	**3**	**4**	**5**	8. How does the child respond when asked to wait and listen to directions for a behavior, task, or activity?
**1**	**2**	**3**	**4**	**5**	9. How does the child respond when verbally or non-verbally asked to stop a behavior, task, or activity that is inappropriate at the time, distressing, bothering, inconveniencing, or endangering someone else?
**1**	**2**	**3**	**4**	**5**	10. How does the child respond when asked to end a challenging behavior, task, or activity?
**1**	**2**	**3**	**4**	**5**	11. How does the child respond when asked to end a behavior, task, or activity being done with a partner or small group?
**1**	**2**	**3**	**4**	**5**	12. How does the child respond when asked to end a behavior, task, or activity being done with a large group or the whole class?

Source: Boyer (2022)

## Appendix B

### Rating Guide for the SASEC

A total score near 24 is typical for 3- to 5-year-olds. Choose the rating number based on the keyword and description that best characterizes how the child typically self-regulates in the situation described by the SASEC item:

**Table:**

**1** Never/ Almost Never	The child will *never* or *almost never* self-regulate in this situation*.* The child remains uninvolved or uncommitted unless it suits her/him. The child may be with someone, in which case that someone will have to do or try to do the behavior, task, or activity *for* the child. Alone or with someone, the child is *distant* (quiet, avoiding eye contact), *reluctant* (walking away, throwing/misusing objects, playing with other materials for a short time, or doing other activities), or *resistant* (screaming, yelling, kicking, constantly moving one or more body parts, working against the behavior, task, or activity, or telling others how to do things).
**2** Infrequently/ Occasionally	The child will *infrequently* or *occasionally* self-regulate in this situation*.* The child may be able to do the behavior, task, or activity but will typically *wait* for someone to *help* them or urge them through repeated modelling or pointing or physically ushering the child. Someone may praise parts of the behavior, task, or activity that the child is willing to do, but *hands-on or direct assistance* is typically required.
**3** Sometimes/ About Half the Time	The child will self-regulate *sometimes* or *about half the time* in this situation. Typically, the child can do the behavior, task, or activity but may not. The child may only do the behavior, task, or activity if someone *explains or models* the expected behavior, task, or activity, and then *convinces* the child with encouragement such as praise, suggestions, or expressions of concern for their well-being while the child is doing the behavior, task, or activity.
**4** Often/ Frequently	The child will *often* or *frequently* self-regulate in this situation*. Typically*, the child will do what is asked and stay with the behavior, task, or activity until it is done. The child may occasionally check with someone or may need occasional adult supervision or reminders about the behavior, task, or activity.
**5** Always/ Almost Always	The child will *always* or *almost always* self-regulate in this situation. *Typically*, the child will do what is asked. The child may ask questions that help with finishing a behavior, task, or activity *because* the child knows *what* is expected but may need some guidance on *how* to do what is expected. The child may *seek* out opportunities to *talk to* an adult about the behavior, task, or activity to gain or maintain positive interaction or to show that they understand what to do or how to do it.

Source: Boyer (2022)

## Code Availability

N.A.
